# Recurrent Breast Cancer in a Patient with a Ventriculoperitoneal Shunt

**DOI:** 10.1155/2015/659395

**Published:** 2015-01-08

**Authors:** Libby R. Copeland-Halperin, Robert A. Cohen

**Affiliations:** Department of Surgery, Inova Fairfax Hospital, 3300 Gallows Road, Falls Church, VA 22042, USA

## Abstract

We report a case of a patient with recurrent infiltrating ductal carcinoma of the breast encasing a ventriculoperitoneal shunt. We also review the current literature regarding reports of breast malignancy around a ventriculoperitoneal shunt, as well as the potential relevance of such shunts to the preoperative evaluation and management of patients with breast cancer.

## 1. Introduction

**Figure 1:**
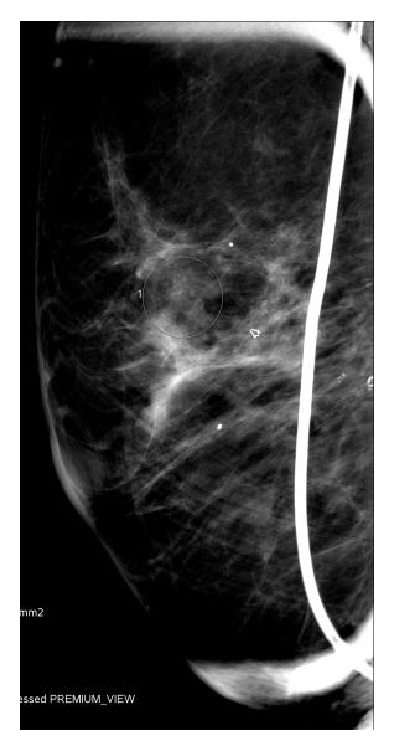
2010 Mammogram. Indeterminate calcifications in right 12:00 retroareolar breast.

**Figure 2:**
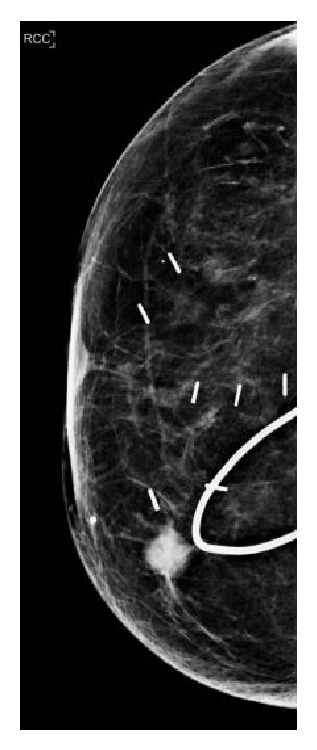
2013 Mammogram. New highly suspicious breast mass at right 1:00 position.

**Figure 3:**
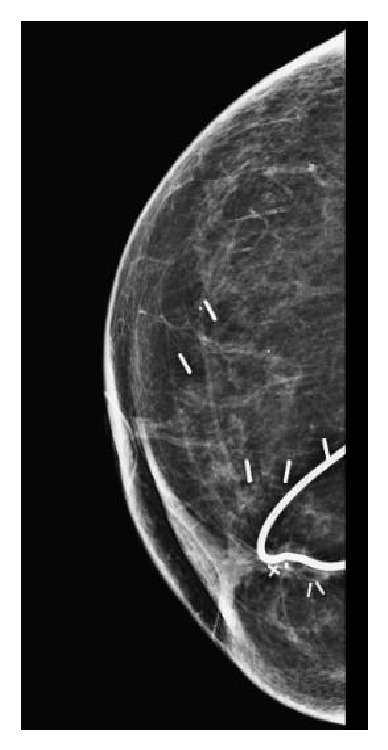
2014 Mammogram. Stable radiopaque wire overlying right breast; interval postlumpectomy changes without malignancy.

A 74-year-old woman with ventriculoperitoneal (VP) shunt installed to relieve hydrocephalus following subarachnoid hemorrhage in 2008 presented 2 years later with grade 2 central ductal carcinoma in situ of the central right breast and underwent stereotactic biopsy and partial mastectomy (Figure 1). She returned 3 years later with T1c N0 M0, grade 3 estrogen receptor (ER) positive, progesterone receptor (PR) positive, HER2/neu negative, and Ki-67 20–25% infiltrating ductal carcinoma of the superior-medial quadrant of the right breast and underwent additional partial mastectomy and sentinel node biopsy followed by chemotherapy and radiation therapy. During the procedure, the VP shunt was embedded in the involved region, requiring dissection around the shunt. The malignancy extended within 1 mm of the caudal margin, but additional excision yielded a specimen free of histologically evident residual disease (Figure 2). Although interim mammography was not concerning (BIRADS 2), clinical examination a year later identified a firm nodule at the site of lumpectomy. Ultrasonography revealed a hypoechoic nodule, biopsy of which contained recurrent invasive ductal carcinoma with lobular features (Figure 3). After extensive discussion of the available options, the patient elected another partial mastectomy, during which cancer was encasing the shunt requiring careful dissection to peel the specimen off the external wall of the shunt. Histological examination revealed Nottingham grade 2 invasive ductal carcinoma with focal lobular features, ER positive, PR negative, HER2/neu negative, and Ki-67 24% moderately differentiated infiltrating ductal carcinoma in the superior-medial quadrant of the breast at the deep inferior margin. After discussion with the patient, presentation at tumor board, and meeting with her oncologist, the patient elected to not undergo a mastectomy or shunt replacement and her endocrine therapy was switched from Letrozole to Faslodex.

## 2. Discussion

Although breast cancer and VP shunts are commonly encountered as separate entities, a review of the English literature revealed previous reports of malignancies of the breast surrounding such a shunt in only 4 cases. In one report, the tumor encased the VP shunt of a 67-year-old woman with a grade 1 infiltrating carcinoma who underwent complete local excision, sentinel node biopsy, and simultaneous rerouting of the shunt and there was no evidence of metastasis. The authors reported that she made an uneventful recovery postoperatively and that the shunt continued to function properly [1].

In another, a large malignant breast mass compressed the shunt to the point of obstruction causing hydrocephalic symptoms [2]. During modified mastectomy, the shunt was found kinked as it coursed through the mass, and the catheter was relocated to the opposite side. The postoperative course was uneventful.

The third report involved an 88-year-old woman with multicentric lobular breast cancer involving a VP shunt without evidence of obstruction [3]. The patient elected partial mastectomy and the mass was resected around the shunt. There was dermal infiltration by tumor around the shunt site and the margins of excision were positive for residual malignancy, raising the possibility that the shunt might have served as a nidus for synchronous metastasis.

The 4th case involved a 52-year-old woman who presented with a VP shunt and a 5 cm subareolar mass [4]. Although the mass did not surround the shunt, it was necessary to dissect around it during modified radical mastectomy. The patient made an uneventful recovery and had normal shunt function postoperatively.

The current standard of care for recurrent infiltrating carcinoma includes mastectomy, but the patient we encountered elected partial mastectomy. Although the margins were negative for residual malignancy at the time of initial resection, recurrence after a relatively short period of time postoperatively raises the possibility that tumor cells might have seeded the shunt surface and, given previous ipsilateral malignancy, leads us to speculate that replacing (rather than repositioning) the shunt might have reduced the risk of recurrent tumor; however, the patient did not wish to undergo this procedure or mastectomy. The impact of the shunt on the risk of local recurrence following intraoperative radiation is also unclear.

## 3. Conclusion

We report the case of a patient with recurrent infiltrating ductal carcinoma encasing a VP shunt despite interim chemotherapy and radiation therapy. This case raises a number of issues, including the potential relevance of such shunts to the preoperative evaluation and management of patients with breast cancer, given the anatomical course of the shunt proximate to the malignancy. The implications extend to needle localization and core biopsy procedures, which must avoid violating the integrity of the shunt, as well as the possibility that these conduits might play a role in the path or risk of extension of malignancy. Preoperative neurosurgical collaboration may be warranted to coordinate exchange or repositioning of the shunt at the time of mastectomy. Further research is needed to assess the impact of radiation on shunt function and clinical outcomes.
